# Cardiac Aspergilloma: A Rare Case of a Cardiac Mass Involving the Native Tricuspid Valve, Right Atrium, and Right Ventricle in an Immunocompromised Patient

**DOI:** 10.1155/2018/6927436

**Published:** 2018-01-29

**Authors:** Christina S. Chen-Milhone, Kalyan Chakravarthy Potu, Sudhir Mungee

**Affiliations:** ^1^Department of Internal Medicine and Pediatrics, University of Illinois College of Medicine at Peoria, Peoria, IL, USA; ^2^Department of Cardiology, University of Illinois College of Medicine at Peoria, Peoria, IL, USA

## Abstract

*Aspergillus* can cause devastating opportunistic infections in immunocompromised patients. Rarely does this fungus invade the heart, and when it does, survival is especially poor despite optimal medical and surgical treatment. We report a case of cardiac aspergilloma with involvement of the tricuspid valve and both the right atrium and ventricle found on a transthoracic echocardiogram in an immunocompromised patient after developing atrial fibrillation with rapid ventricular rate. The findings from this case suggest that early clinical suspicion is critical in early diagnosis and thus early treatment.

## 1. Introduction

*Aspergillus* is a common fungus in the environment that can cause fatal diseases in humans. The *Aspergillus* spores are easily inhaled, and immunocompromised individuals are known to be more susceptible to its disease. Acute invasive aspergillus of the heart is particularly uncommon. *Aspergillus* endocarditis accounts for 20–25% of all cases of fungal endocarditis, and fungal endocarditis only comprises <2% of all cases of endocarditis [1, 2]. Invasive aspergillus involving heart valves and chambers is even rarer with only a handful of cases reported in the literature. In this report, we present a case of cardiac aspergilloma involving the native tricuspid valve and both the right atrium and ventricle that initially presented as a cardiac mass in an immunocompromised patient.

## 2. Case Report

A 62-year-old male presented to an outlying hospital with a one-month history of productive cough, shortness of breath, and intermittent fevers, and a syncopal episode one week before. His past medical history was significant for hypertension, prostatic adenocarcinoma with bone metastasis, atrial fibrillation, and bilateral pulmonary embolism. The prostatic adenocarcinoma was diagnosed 11 months before, and he developed the bilateral pulmonary embolism approximately 8 months before. He completed 40 cycles of radiation, and medications included Zytiga in conjunction with prednisone, Lupron, and Eliquis. He was a former smoker and smoked a maximum of 5 cigarettes daily for 35 years. His social history was otherwise negative for high-risk sexual behavior, intravenous (IV) drug use, recent travel, and occupational exposure. He was subsequently admitted with a diagnosis of anemia with hemoglobin of 7.1 g/dL and sepsis secondary to presumed pneumonia.

**Figure 1:**
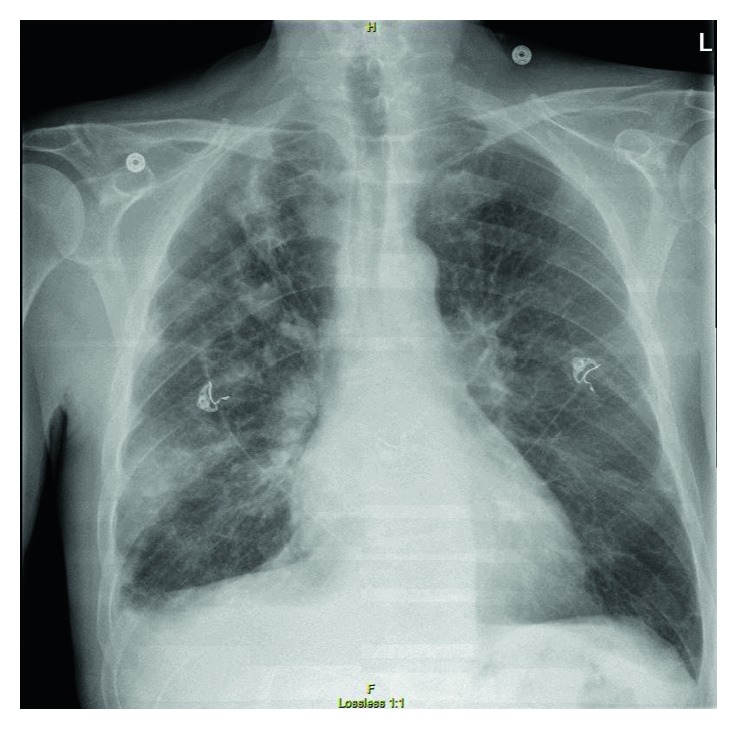
CXR showing multiple right pulmonary nodules.

On admission, the patient received 4 units of packed red blood cells for anemia. The patient was also noted to have thrombocytopenia with platelet count of 48,000/mcL; thus, Eliquis was held. Chest X-ray revealed a 7.4 cm opacity in the right lung base as well as multiple right pulmonary nodules (Figure 1). A CT chest showed similar findings with the addition of extensive bone metastasis. The patient was started on vancomycin and Zosyn for sepsis while awaiting blood culture results. Infectious workup was positive for *Aspergillus fumigatus* IgG, *Aspergillus galactomannan* antigen (Ag), *Histoplasma* Ag, Fungitell, and *Blastomyces* antibody (Ab). Human immunodeficiency virus (HIV), herpes simplex virus (HSV), and influenza A and B serologies were negative. Blood cultures were negative for viruses, bacteria, and fungi. Infectious diseases were consulted. The patient subsequently underwent a bronchoscopy, with lavage specimens returning positive for *Aspergillus* Ag but negative for pneumocystis pneumonia, HSV, bacteria, fungus including *Histoplasma*, and malignant cells. The patient was then diagnosed with presumed subacute invasive pulmonary aspergillus. Antibiotics were then discontinued, and the patient was discharged with voriconazole for a 6-week duration.

**Figure 2:**
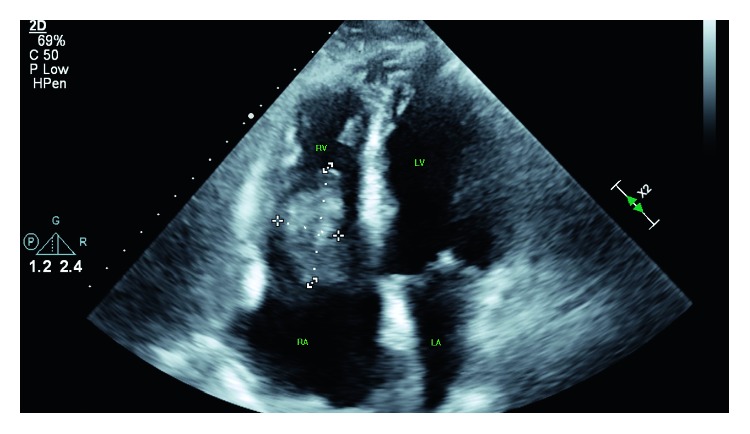
Transthoracic echocardiogram showing a mass involving the tricuspid valve and both the right atrium and ventricle.

**Figure 3:**
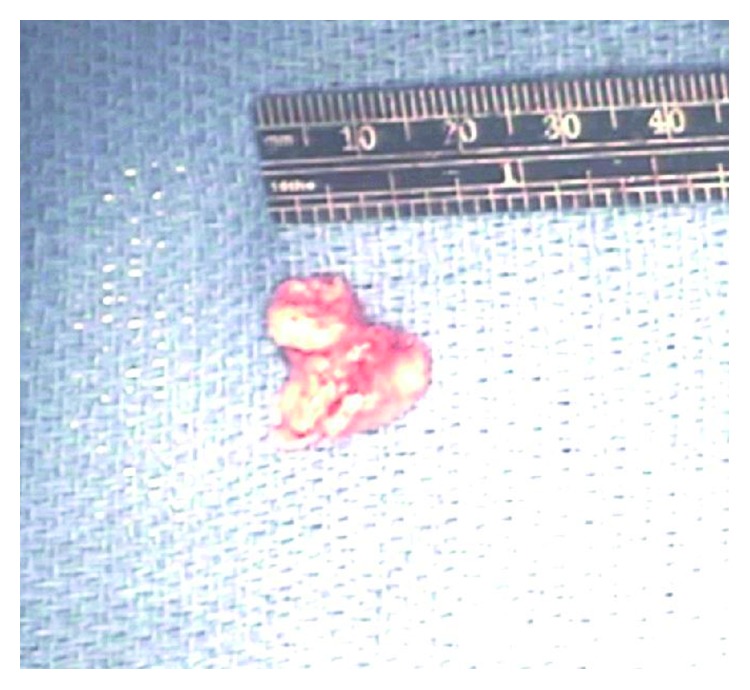
Right atrial and tricuspid ventricular mass.

**Figure 4:**
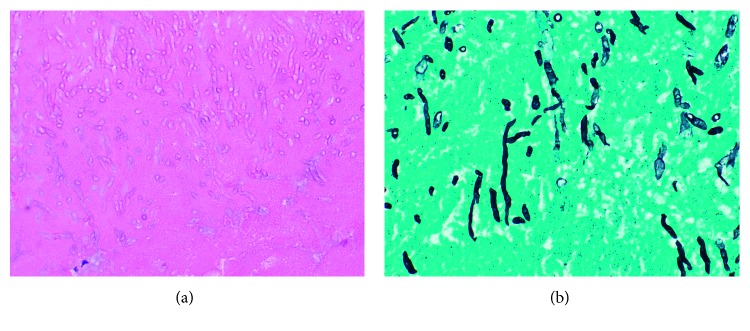
Specimen of the mass with H&E stain (a) and GMS stain highlighting fungal elements (b).

Two days after discharge, he was admitted for atrial fibrillation with rapid ventricular rate. A transthoracic echocardiogram (TTE) was performed and showed a highly mobile 5 cm mass involving the tricuspid valve with variable involvement of the right atrium and right ventricle (Figure 2). The ejection fraction was preserved. Infectious diseases and oncology were then consulted. The patient remained on voriconazole, and Zytiga was discontinued as his prostate cancer was castration sensitive, per oncology. CT surgery was consulted and performed sternotomy with resection of the mass, portions of the right atrium and ventricle, and tricuspid valve on day 2 of admission. The excised mass was pale yellow and pink, irregularly shaped, fragmented, bulky, and approximately 3 × 2 × 1.3 cm (Figure 3). Pathology from the mass and tricuspid valve showed necrotic debris with acute inflammation and abundant fungal hyphae (Figure 4). Cultures obtained from tissue in the operating room were positive for *Aspergillus fumigatus* and negative for bacteria and viruses.

The patient did well postoperatively. He was extubated to nasal cannula within 12 hours; however, he remained on pressor support. Voriconazole was continued. On day 4 of admission, he developed septic shock, renal failure with oliguria and acidosis, and shock liver requiring increased pressor support. He continued to deteriorate the next day requiring endotracheal intubation and ventilator support. On day 5 of admission, the patient developed ventricular tachycardia followed by pulseless electrical activity. Shortly thereafter, he was pronounced dead. The cause of death was invasive aspergillosis-induced septic shock with multiorgan failure.

## 3. Discussion

Aspergilloma of the heart is a rare disease that carries a very poor prognosis. On review of the current literature, only a small number of cases have been reported. These cases include an invasive aspergillus of the interventricular septum in an allogenic stem cell transplant patient [3], aspergilloma involving all four chambers of the heart [4], aspergillosis presenting as a pedunculated mass with multiple nodules on the left ventricular wall in an acute lymphoblastic leukemia patient [5], a large right ventricular aspergilloma in a young man on chemotherapy for acute myelogenous leukemia [6], an incidental finding of a tricuspid valve aspergilloma after abdominal surgery [7], and the three cases described in the correspondence by Wiwanitkit and Wiwanitkit [8]. Of note, all except for one of the aforementioned cases resulted in death within days to weeks after diagnosis.

Fungal endocarditis alone, without involvement of the surrounding cardiac structure, is associated with a mortality rate of at least 50% with an average survival period of 11 days [9]. Fungal endocarditis has also recently been shown to have increasing prevalence with medical and surgical advancements [1, 2]. Historically, the main risk factors for developing fungal endocarditis included previous valve surgery, history of prosthetic valve endocarditis, prolonged use of antibiotics, rheumatic heart disease, and IV drug use [1]. Most recent studies suggest that these characteristics have changed and that fungal endocarditis now more commonly affects immunocompromised patients, patients with prosthetic valves and central venous catheters, and those on broad-spectrum antibiotics [1, 2]. Of note, *Aspergillus* endocarditis is mostly found on aortic or mitral prosthetic valves [10] and has a mortality rate of approximately 80% [2].

Prolonged fever, as seen in the described case, is the most common presenting feature; however, some patients may present with new or changing heart murmur, chest pain, cough, dyspnea, hemoptysis, weakness, or generalized pain. Hemorrhagic black skin lesions have been reported in *Aspergillus* endocarditis [11]. Such symptoms in combination with leukocytosis, anemia, and thrombocytopenia may also be suggestive of fungal septicemia. As discussed earlier, an immunocompromised state, neutropenia, recent history of long-term central venous catheter, prosthetic heart valve, steroid use, prolonged antibiotic therapy, recent prolonged hospitalization, parenteral nutrition, immunocompromised status, and IV drug use predispose patients to an invasive fungal infection.

With the diverse symptoms and risk factors, the diagnosis of fungal endocarditis is rather challenging and can be easily delayed without a high clinical suspicion, especially given that fungal pathogens are slow growing. Diagnosis is further complicated as the majority of fungi are not detected in blood cultures. *Aspergillus*, in particular, is positive in 4–30% of blood cultures in cases of *Aspergillus* endocarditis [2, 12]. Some reviews have also suggested testing for galactomannan antigen, which is released when *Aspergillus* proliferates [1, 12]. TTE is a valuable diagnostic tool in diagnosing endocarditis and has been shown to identify 89% and 76.5% of the vegetations in native and prosthetic valves, respectively [2]. Although vegetations seen on TTE may not distinguish between bacterial versus fungal etiology, fungal vegetations are typically characterized as a large, bulky, highly mobile mass, as seen in the described case, and cause an increased risk of embolic events, more commonly affecting the brain. Still, many cases of fungal endocarditis are diagnosed during surgery or autopsy.

Upon diagnosing fungal invasion of the heart, a multidisciplinary team involving cardiology, infectious diseases, and cardiovascular surgery is necessary. Most recent reviews as well as Infectious Diseases Society of America (IDSA) guidelines favor a combined medical and early surgical intervention for treatment of *Aspergillus* endocarditis, pericarditis, and myocarditis. Surgical valve replacement in addition to systemic antifungal therapy was noted to be pertinent to survival due to poor prognosis and risk of relapse in patients receiving only pharmacological therapy, decreased effect of antifungal medication on fungal vegetations, large vegetations possibly compromising cardiac function, and the risk of embolic events [1, 12]. Per IDSA, the recommended initial antifungal agents are voriconazole or liposomal amphotericin B 3–5 mg/kg/day, followed by lifelong antifungal treatment postsurgical valve replacement [13]. Amphotericin B is preferred for patients with hepatic insufficiency [12]. Some have proposed using caspofungin in addition to voriconazole and amphotericin [14]; however, the use of combining antifungal therapies has not been shown to be superior to monotherapy [13]. In the described case, the patient was already on voriconazole and underwent surgical resection.

The differential diagnosis for the cardiac mass beyond a fungal etiology includes thrombus, primary cardiac tumor, myxoma, metastatic lesion, and lymphoma. Though the patient had new-onset atrial fibrillation, a thrombus was unlikely as he had been on apixaban for approximately 6-7 months. Prostate cancer does not typically metastasize to the heart; thus, metastasis was also unlikely. Given the patient's immunocompromised state, being on steroids, and recent diagnosis of invasive pulmonary aspergillosis, the clinical suspicion for a fungal mass was high. Hematogenous spread of invasive pulmonary aspergillus was considered in the development of cardiac aspergilloma. The spread of invasive pulmonary aspergillus to the heart is very rare and is associated with a high mortality estimated to be as high as 80–90% [15], which is approximately the same mortality rate of having *Aspergillus* endocarditis alone, despite aggressive pharmacological and surgical interventions [2]. However, the hematogenous spread of invasive pulmonary aspergillus would be expected to invade the left cardium rather than the right, as seen in our patient.

In considering the described case, prior to discovering the cardiac aspergilloma, the patient was already on antifungal therapy. Furthermore, once a fungal mass was suspected, the patient underwent surgery and continued antifungal treatment as per recommendations. The overall outcome, nonetheless, was grim.

Cardiac aspergilloma remains a rare yet deadly disease that carries a high mortality rate despite aggressive pharmacological and surgical interventions. Decreasing the mortality rate remains a challenging task, given the nature of the disease and diagnostic barriers to detecting the disease early on. While innovative molecular methods can detect the diagnosis sooner than was previously possible, clinical suspicion remains the key to diagnosing the disease. Early clinical suspicion will lead to early treatment, and earlier treatment will presumably result in decreased mortality.
